# Radiography using cosmic-ray electromagnetic showers and its application in hydrology

**DOI:** 10.1038/s41598-022-24765-7

**Published:** 2022-11-27

**Authors:** A. Taketa, R. Nishiyama, K. Yamamoto, M. Iguchi

**Affiliations:** 1 Earthquake Research Institute, The University of Tokyo, Tokyo, 113-0032 Japan; 2 Disaster Prevention Research Institute, Kyoto University, Kagoshima, 891-1419 Japan

**Keywords:** Experimental particle physics, Particle astrophysics, Geophysics, Hydrology

## Abstract

In-situ measurements of soil water content provide important constraints on local/global hydrology. We demonstrate that the attenuation of the underground flux of cosmic-ray electromagnetic (EM) particles can be used to monitor the variation of soil water content after rainfalls. We developed a detection system that preferably selects EM particles by considering the coincidence of distant plastic scintillators. The calibration test beneath the water pool revealed that the count rate decreased by 0.6–0.7% with a 1 cm increase in the water level. The field measurement performed in the horizontal tunnel showed that the count rate dropped according to 48-h precipitation, after correcting the effects originating from atmospheric and water vapour pressures. These characteristics were confirmed using dedicated Monte Carlo simulations. This new method is called cosmic electromagnetic particle (CEMP) radiography.

## Introduction

The nature of cosmic rays has been investigated by many physicists in the last hundred years^1,2^. Meanwhile, researchers have been attempting to utilize them in research fields of Earth sciences. Secondary cosmic rays on Earth’s surface are practically classified as (i) muons, (ii) electromagnetic particles (electrons, positrons, photons), (iii) hadronic particles (protons, neutrons, and mesons), and (iv) neutrinos. The characteristics of muon components (component i) have been studied by many physical experiments at various locations^3^, as muons could be the main source of the background noise in underground experiments. Key factors controlling the temporal variation of muon flux have been reported in much literature (e.g. barometric effects^4–6^, mid-altitude temperature effects^7–9^, tidal effects in ocean bottom measurements^10^). In the last decade, radiography using the high penetration power of the muons has been highlighted (muography) and applied to the inspection of volcanoes^11–19^, glaciers^20^, seismic faults^21,22^, mineral deposits^23^, archeological sites^24^, etc. Neutrino radiography (component iv) is considered to be a promising probe for far deep structures, including the core of the Earth^25,26^ because of their extremely high penetration power. In addition, increasing attention is paid to measurements of cosmic-ray-induced neutrons (component iii) as a useful tool for monitoring snow depth^27–29^ and soil moisture content^30–32^. Looking at geochemistry, the dating methods by cosmogenic nuclide^33,34^ (e.g. ^10^Be, ^26^Al, ^36^Cl) could be regarded as a geoscientific application of cosmic rays, where muons and neutrons are involved in the production of those nuclei. Among the classifications above, only electromagnetic particles (component ii) have not been largely used in specific geoscientific applications, except for several works pointing out a possible correlation between electromagnetic particles and thunderstorms^35^. Here, the present study proposes a radiographic use of electromagnetic particles in hydrological studies.

**Figure 1:**
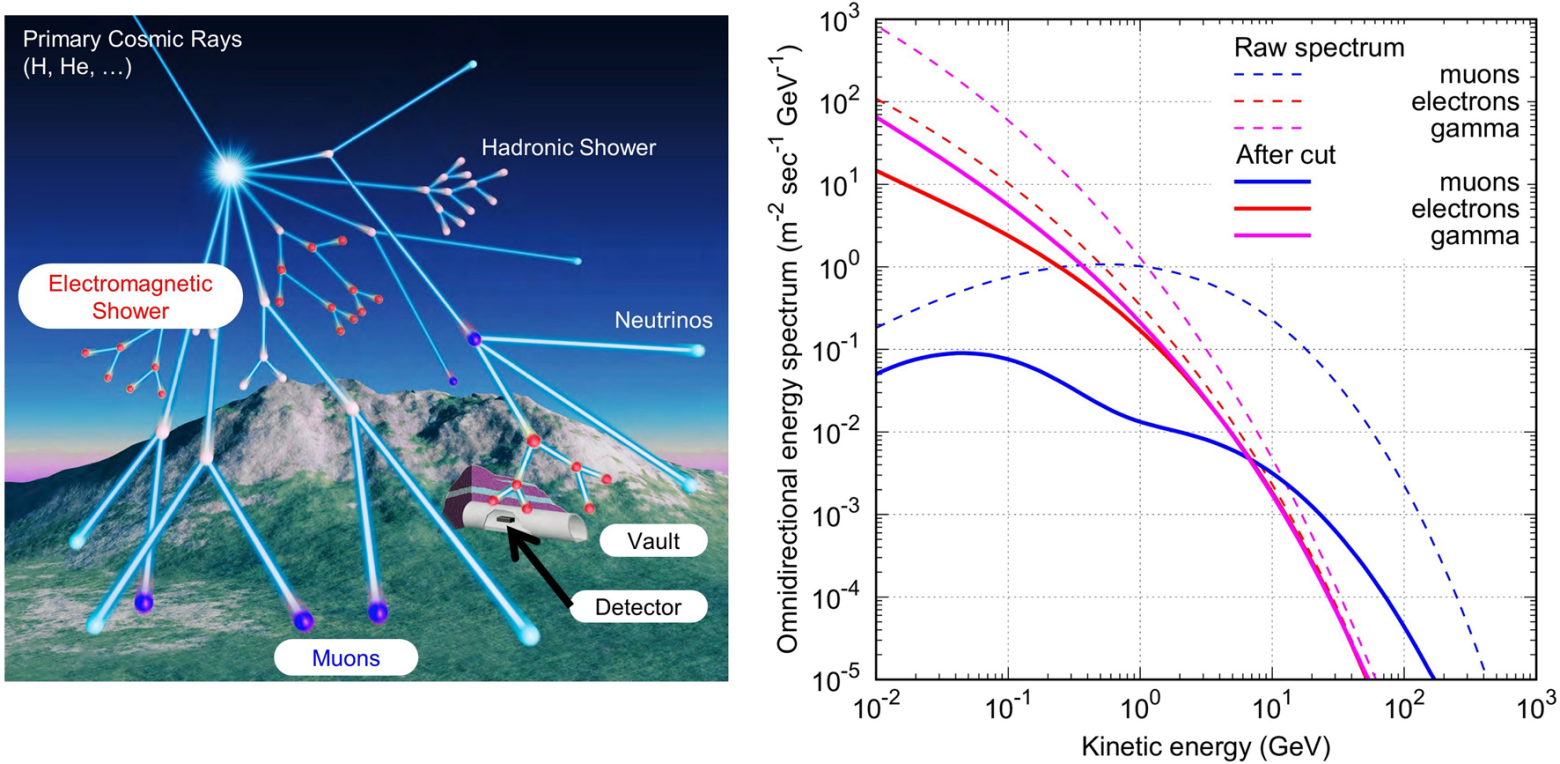
Cosmic rays and energy spectrum. (Left) Development of cosmic ray showers in the atmosphere, with electromagnetic (EM) showers indicated by red points. (Right) energy spectrum of secondary cosmic rays on the Earth’s surface, calculated using COSMOSX simulator (see “Method” section). The dashed lines represent the raw spectra of muons, electrons, and gamma rays. The solid lines are the spectra of the same particles but for cases when multiple particles arrive into 1 m × 1 m region simultaneously (within 500 nsec). The original version of the left figure was created by Nariyuki Yoshihara (Photon Create) and modified by R.N and A. T.

Electromagnetic showers consist of electrons, positrons, and photons (referred to as EM particles hereafter in the manuscript). They are predominantly produced from cascades initiated by the decay of neutral and charged mesons in the upper atmosphere. Figure 1 presents a schematic illustration of an extensive air shower initiated by a high-energy cosmic ray, which first generates high-energy mesons. The EM particles are mainly generated by π^0^ mesons. A π^0^ meson decays into two photons (γ), interacts with atmospheric nuclei, and produces further pairs of e^+^ and e^-^. The produced electrons subsequently emit γ on interaction with another nucleus, resulting in the development of an extensive air shower^36^. Figure 1 shows the simulated energy spectra of EM particles (red) and muons (blue) at sea level. Although muons are the most numerous charged particles above 1 GeV, EM particles dominate in the sub-GeV range. These sub-GeV EM particles can be feasible probes for tiny mass fluctuations near the surface of the Earth because their energy loss is greater than that of muons by a factor of up to ten for the same kinetic energy^37,38^. Monitoring the temporal variation in the rates of EM particles arriving underground detectors would yield information on the mass variation in the overburden soil. Among several possible applications, the present study focused on monitoring the moisture content in the soil.

**Figure 2:**
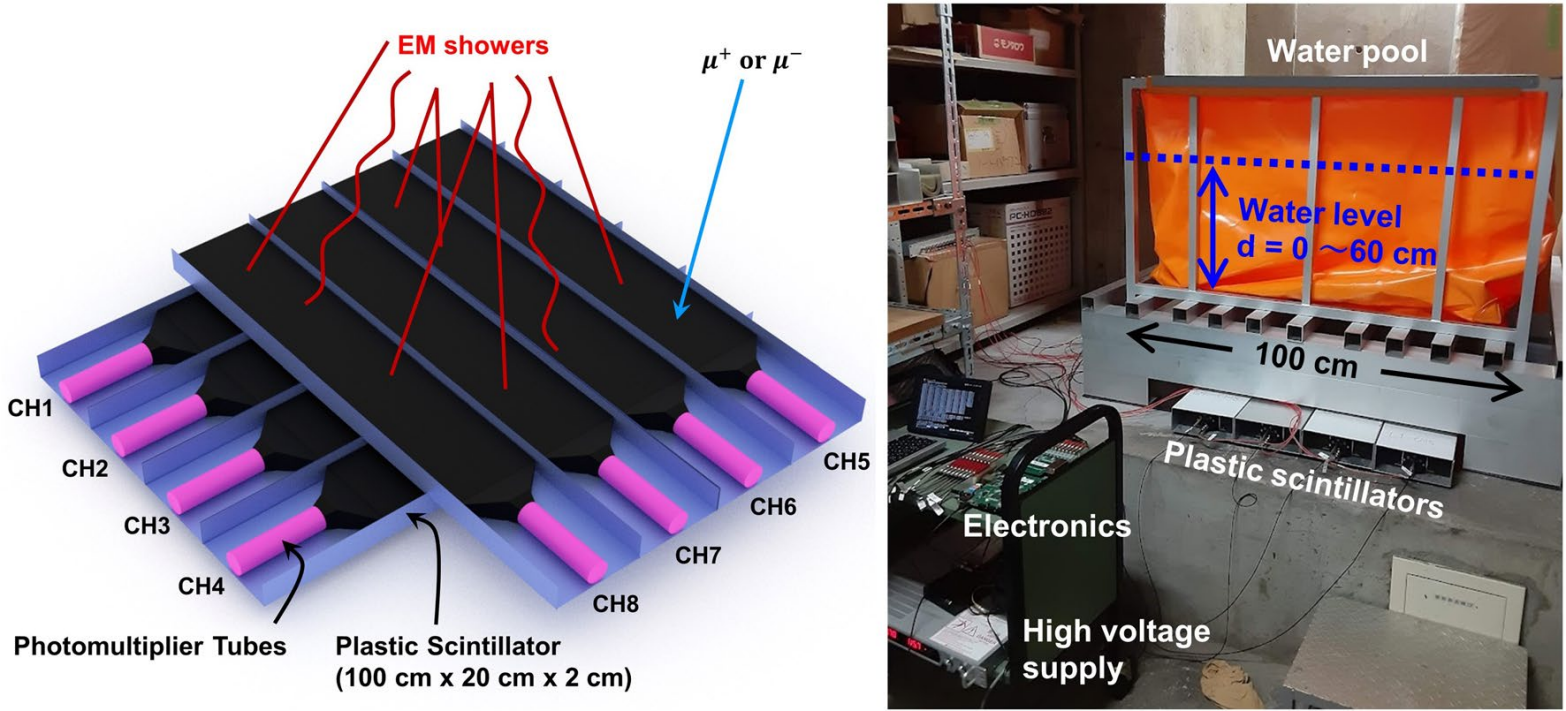
Detector setup. (Left) Schematic illustration of the detection system for EM showers. (Right) geometry of the water pool calibration experiment in the laboratory.

This idea necessitates particle detectors that can preferably discriminate EM particles from other cosmic-ray components. Such selection is possible by focusing on the avalanche nature of EM showers; when EM particles fall to the ground, they are presumably not alone. In other words, while most muons hit the detector alone, EM particles can hit multiple distant particle detectors simultaneously by optimising the detector arrangement. Therefore, it is possible to statistically enhance the events due to EM particles by taking the coincidence of detectors within a certain spatiotemporal range. By choosing the cases where multiple particles arrive at 1 × 1 m region simultaneously, the contribution of EM particles is enhanced compared to muons (solid curves in Fig. 1). This method does not require strong magnets or thick absorbers for particle identification. This concept was realised by developing a new detection system with plastic scintillators (PS), photomultiplier tubes (PMT), and readout electronics. The system consists of upper and lower sensitive layers. Each layer was segmented into four channels of PS (100 × 20 × 2 cm) + PMT (Fig. 2). The scintillation light is converted into electric signals by the PMTs and collected by the readout electronics. We defined an EM event as coincidental hits of at least two PMTs in both the upper and lower layers. The technical specifications and details of the system are described in the methods section.

We performed measurements with the new detection system at two locations: (1) beneath a water tank in laboratories located on different floors (Tokyo, Japan; see Fig. 2), and (2) in a horizontal vault (Kagoshima, Japan; see Fig. 4). The former is aimed at evaluating how the EM flux changes according to the variation in the overburden water and concrete, and verifying such changes with a dedicated Monte Carlo (MC) simulation. The latter setup verifies that the proposed method is sensitive to variations in soil moisture content after rainfall in real field conditions.

## Results

### Calibration experiment with water pool

**Figure 3:**
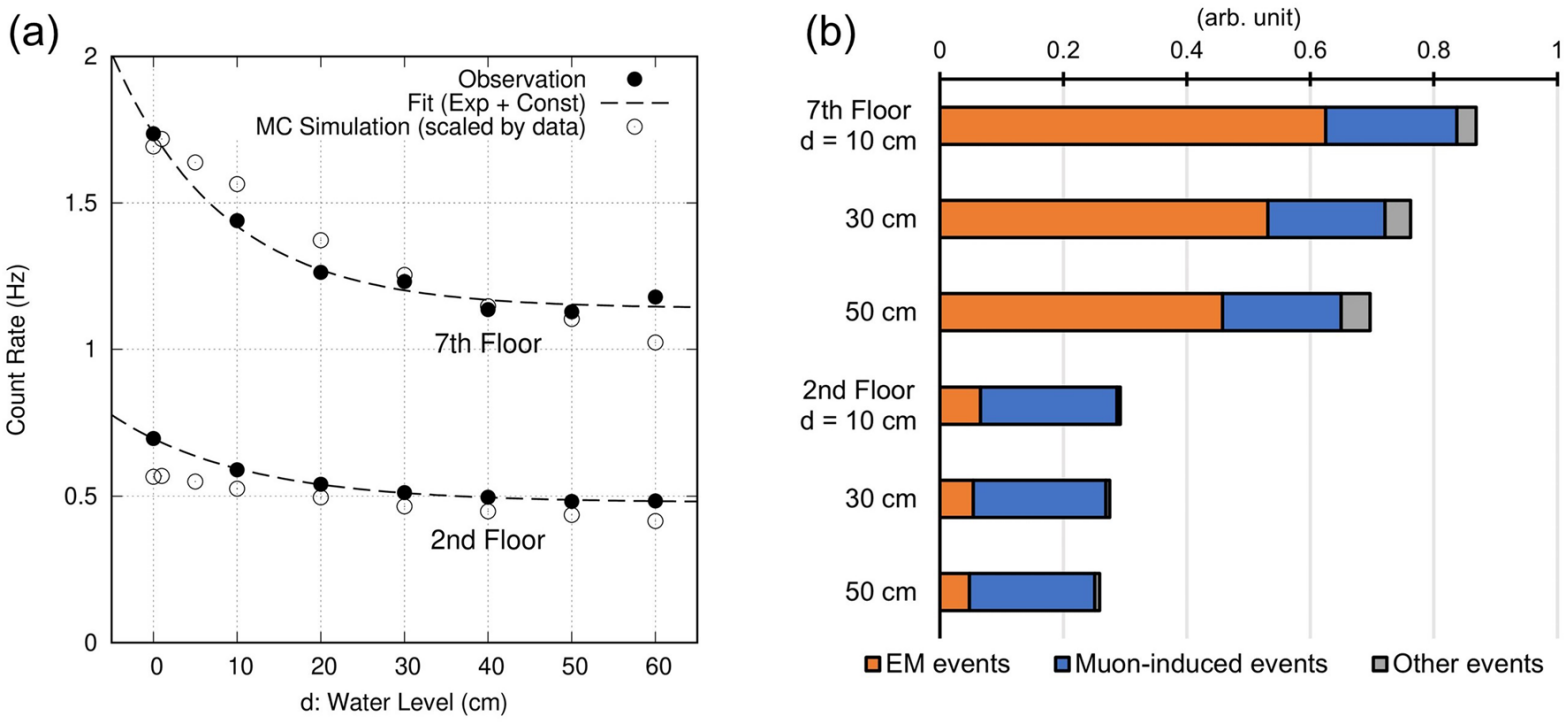
Results of the calibration experiment with the water pool. (**a**) Observed count rate in the calibration test as a function of the water level in the pool (solid circles) together with the simulated count rate (open circles). (**b**) Breakdown of the events from simulation. EM events: coincidental hits of electromagnetic showers; Muon-induced events: coincidental hits where at least one muon is involved; Other events: not classified in the other two categories.

The calibration experiment was performed on the 2nd and 7th floor of Building 2 (8-storey building), Earthquake Research Institute, University of Tokyo (35.7187°N, 139.7597°E, Fig. 2). A water pool was placed 20 cm above the detection system. The water level was changed from 0 to 60 cm in increments of 10 cm. Data were recorded for more than 5 h at each water level. Event selection required detection of signals by at least two PMTs in both the upper and lower detection layers within a time window of 500 ns. The solid circles in Fig. 3a show the count rates of selected events. The observed count rates were compared with simulations (open circles, Fig. 3a) using MC codes (see “Method” Section for details).

The observed count rate gradually decreased with increasing water level. The decrease rates were approximately 0.6%/cm for the 2nd floor and 0.7%/cm for the 7th floor. By fitting the count rate data with exponential + constant curves (dashed curves in Fig. 3a), decay lengths are determined to be $15.8\pm 0.7 \mathrm{cm}$ and $13.3\pm 2.2 \mathrm{cm}$ for the 2nd and 7th floors, respectively. The general characteristics of the count rate were well reproduced by the MC simulation, although the absolute count rate differed by a factor of 1.8. Figure 3b shows a breakdown of the simulated events, which represents the abundance of events originating from EM showers and others. EM showers attenuate greatly with an increase in the water level and thickness of the overburden concrete walls, whereas the occurrence of muon-involved events is nearly constant. Therefore, the decrease in the observed count rate is due to the rapid absorption of EM showers.

### Field verification in the horizontal vault

**Figure 4:**
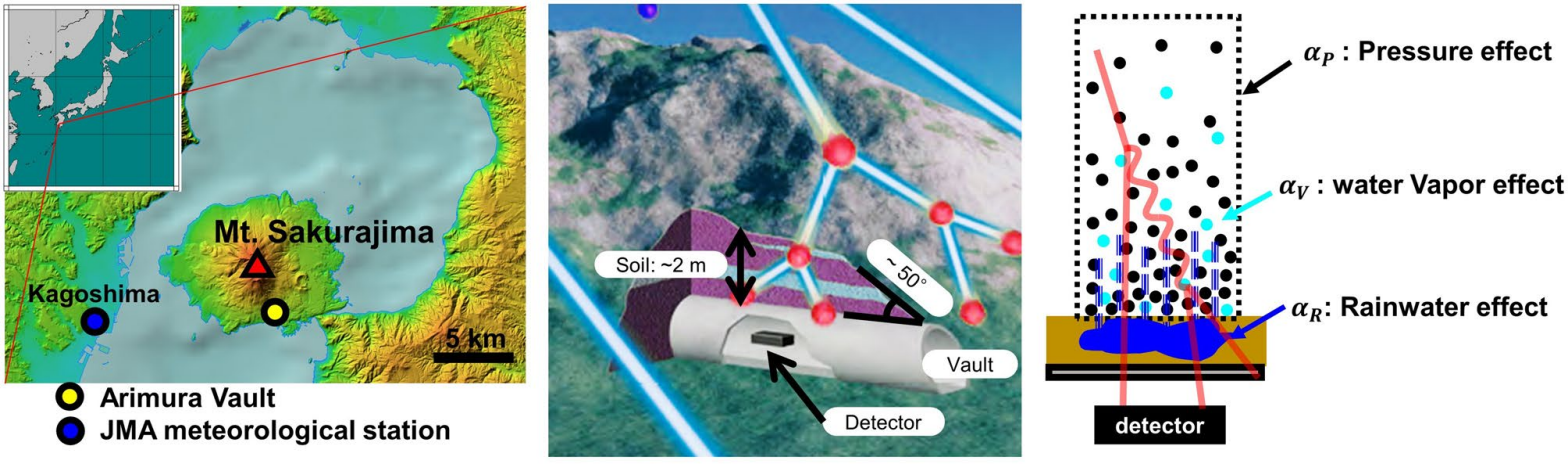
Setup of the field measurement. (Left) Location of Arimura Crustal Movement Observation Vault, Kagoshima, Japan. The basemap is provided by the Geospatial Information Authority of Japan website (GSI, “color elevation map” available at https://maps.gsi.go.jp/). The inset map is generated by the Generic Mapping Tools 5.0^56^. These maps were arranged by R. N. and A. T. with Microsoft Powerpoint software (https://www.microsoft.com/). (Middle) Geometry of the field measurements near the entrance of the horizontal vault. The original version of the figure was created by Nariyuki Yoshihara (Photon Create) and modified by R. N. and A. T. (Right) Schematic illustration of the three effects controlling the count rate (atmospheric pressure effect, water vapour effect, and rainwater effect).

Based on the success of the calibration test, field measurements were performed in a horizontal vault (Arimura Crustal Movement Observation Vault, Kagoshima, Japan; 31.5602°N, 130.6720°E). The detection system was installed near the entrance of the vault (Fig. 4) where the overburden soil was approximately 2 m (dry bulk density of soil is approximately 1.5 g/cm^3^, inferred from Ref.^39^). The annual rainfall is approximately 2200 mm, and the rainiest months are June and July^40^. Measurements were performed intermittently between 2014 and 2019. The total measurement duration was 960 days. The count rate data is available at "supplementary table".

In field measurements, the atmospheric effects on the count rate must be properly corrected before discussing the variation in soil moisture content after rainfall. In this study, we focused on atmospheric pressure and water vapour pressure. First, we took the 48-h average of the atmospheric pressure, the water vapour pressure, and the count rate. Next, we selected the data for the non-rainy period (48-h precipitation = 0 mm) and fit the count rate data using the regression model described below:

1 $$\begin{array}{c}\frac{\Delta N}{\langle N\rangle }={\alpha }_{\mathrm{P}}\Delta P+{\alpha }_{V}\Delta V,\end{array}$$

where $\langle N\rangle$ is the mean count rate of EM events, and $\Delta N$ is the deviation from the mean. $\Delta P$ and $\mathrm{\Delta V}$ are the atmospheric and water vapour pressures, respectively. ${\alpha }_{\text{P}}\, \mathrm{and}\, {\alpha }_{V}$ are the coefficients determined from the regression analysis. The linear assumption employed in the regression analysis is valid because the fluctuation of the cout rate is no more than 5%. The resulting coefficients are as follows:

Atmospheric Pressure effect:

$${\alpha }_{\text{P}}=-0.088\pm 0.008 (\%/\mathrm{hPa})$$

Water Vapor pressure effect:

$${\alpha }_{\text{V}}=-0.019\pm 0.007 \left(\%/\mathrm{hPa}\right),$$

where the goodness of fit was ${\chi }^{2}= 355$ (degrees of freedom = 154). The cross-correlation coefficient between ${\alpha }_{\text{P}}\,\mathrm{and}\, {\alpha }_{V}$ was 0.80.

**Figure 5:**
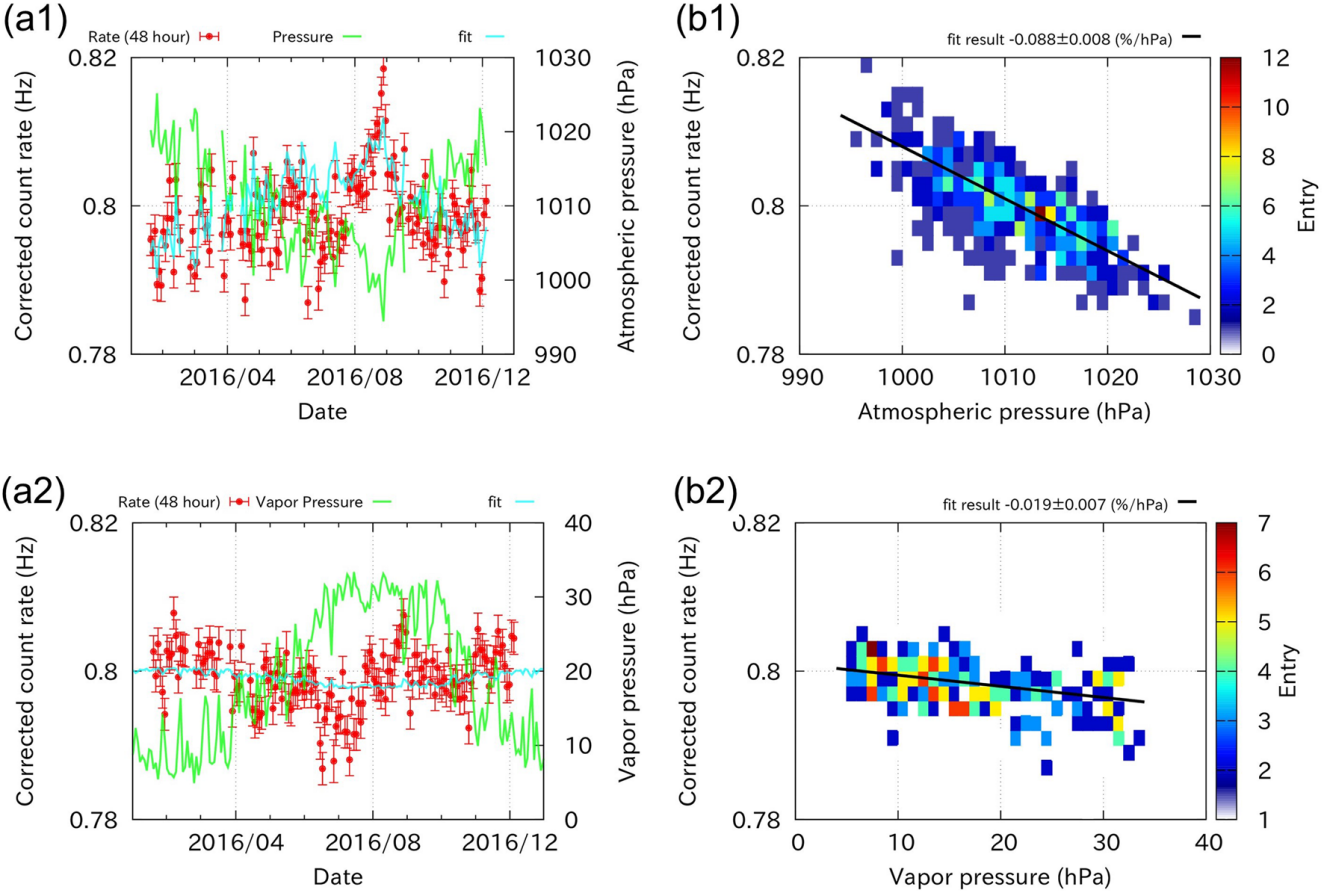
Results of the field measurement and atmospheric corrections. (Top) Effect of the atmospheric pressure on the count rate ((**a1**): the observed count rate after correcting the vapour pressure effect together with the atmospheric pressure, (**b1**): fitting result). (Bottom) Effects of the vapour pressure on the count rate ((**a2**): the count rate after correcting the atmospheric pressure effect together with the vapour pressure, (**b2**): fitting result).

Figure 5a1 shows the temporal variation in the count rate every 48 h observed in 2016 after correction for the water vapour pressure effect. The count rate shows a clear anti-correlation with atmospheric pressure. This is because the molecules in the atmosphere act as absorbers for electromagnetic showers. The regression analysis shows that this pressure effect is a primary term that controls the count rate. This characteristic is consistent with the results of the MC simulation (Fig. 7e). Figure 5a2 shows the count rate after correction for the atmospheric pressure effect. There is still a small anti-correlation with atmospheric water vapour pressure. The corresponding coefficient ${\alpha }_{\text{V}}$ is smaller than ${\alpha }_{\text{P}}$ but is not negligible. Moreover, the simulation also predicts that the count rate would decrease slightly with an increase in the atmospheric vapour pressure (Fig. 7f). This effect is presumably because water molecules in the atmosphere change the stopping power for charged particles (see the “Discussion” section). In fact, the water vapour pressure is strongly coupled to the variation in atmospheric temperature, and the effects of these two factors are difficult to separate. Therefore, in this analysis, we considered only the effects of atmospheric pressure and water vapour pressure.

**Figure 6:**
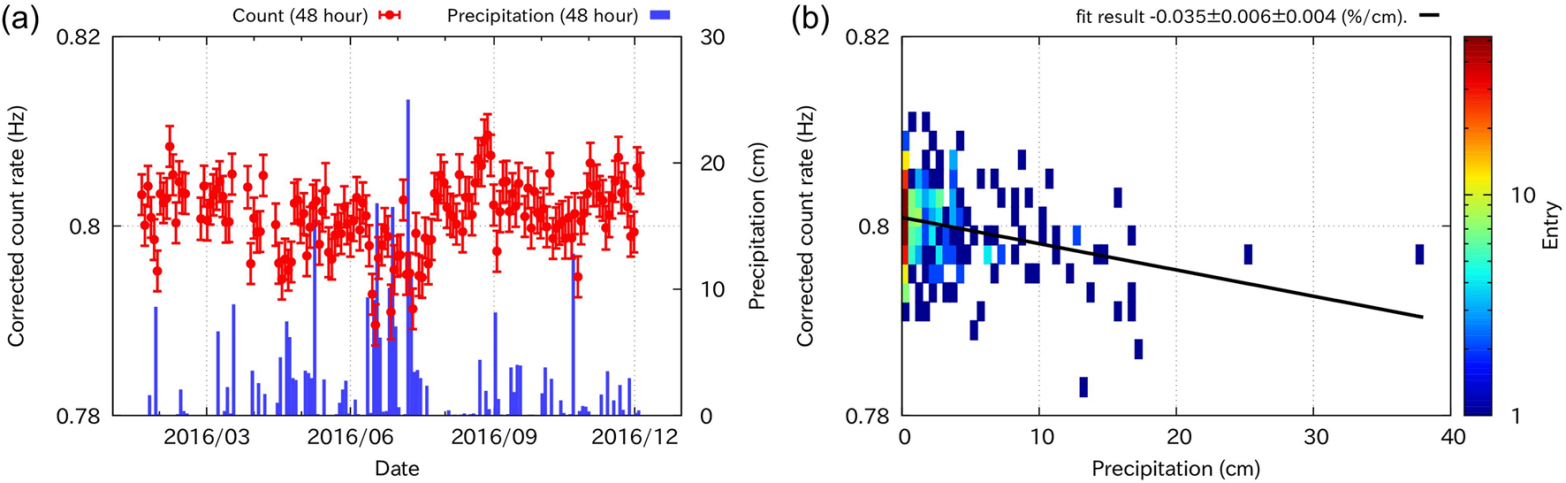
Decrease in EM count rate due to rainfall events. (**a**) Count rate (every 48 h) after correcting the atmospheric effects (see Fig. 5), together with the 48-h-cumulative precipitation. (**b**) Fitting results of the corrected count rate as a function of the precipitation.

Now that the atmospheric effects have been extracted and corrected, we discuss the effect of soil water content. Since we have no in-situ measurements of soil moisture content above the detector, we use the precipitation data as an index of it. According to the past results of in-situ measurements 20 m away from the vault^41^, the volume content of water in Sakurajima soil is typically 10–20% and it rises to ~ 30% immediately after rainfalls and decays gradually. Figure 6a shows the count rate after correcting the atmospheric effects described in the previous paragraph, together with the 48-h-cumulative precipitation. The results showed that sudden drops in the corrected count rate were accompanied by heavy rain events from June to July 2016. For a more quantitative discussion, the corrected count rate was fitted with the 48-h precipitation (Fig. 6b). As a result, the coefficient of the rain effect ${\alpha }_{\text{R}}$ is determined as:

Rainwater effect:

$${\alpha }_{\mathrm{R}}=-0.035\pm 0.006\pm 0.004 (\%/\mathrm{cm}),$$

where the goodness of fit was ${\chi }^{2}= 1081 \left(\nu = 406\right).$ The first error of ${\alpha }_{\mathrm{R}}$ is due to statistical uncertainty, and the second error is due to the uncertainties of the ${\alpha }_{\text{P}}\, \mathrm{and} \ {\alpha }_{\mathrm{V}}$.

## Discussion

The decrease in the count rate accompanied by rainfall is due to rainwater percolating into the soil above the detector (see the geometry of Fig. 4). The observed decrement is proportional to the 48-h-cumulative rainfall with a coefficient ${\alpha }_{R}= -0.035\pm 0.006\pm 0.004 (\%/\mathrm{cm}).$ This effect is independent of the variation in the atmospheric pressure and water vapour pressure because these atmospheric effects were corrected using the data without rainfall. However, the observed precipitation coefficient was smaller than the decrease rate observed in the water pool calibration test (0.6–0.7%/cm, Fig. 3a). This difference up to a factor of 10 can be attributed to the low infiltration capacity of the soil near Mt. Sakurajima volcano ($\sim {10}^{-3}\mathrm{cm}\,{\mathrm{s}}^{-1}$)^39,42^. According to the past in-situ measurements^41^, the increment of the volume water content is no more than 20% even after heavy rainfall events. Another possible reason is that some part of the overburden soil is covered with concrete; the rainwater may not infiltrate into the soil well and run off immediately on the surface of the slope. A comparison between the observation and calibration test implies that the soil above the entrance of the Arimura Vault does not retain much rainwater. This type of hydrological knowledge obtained from EM particle measurements will be useful for correcting seismic and geodetic signals accompanied by movements of underground water. For instance, gravimeters installed for volcano monitoring are disturbed by rainfall and groundwater^43,44^. The hydrological parameters provided by the present study would be useful for understanding and correcting water movements.

**Figure 7:**
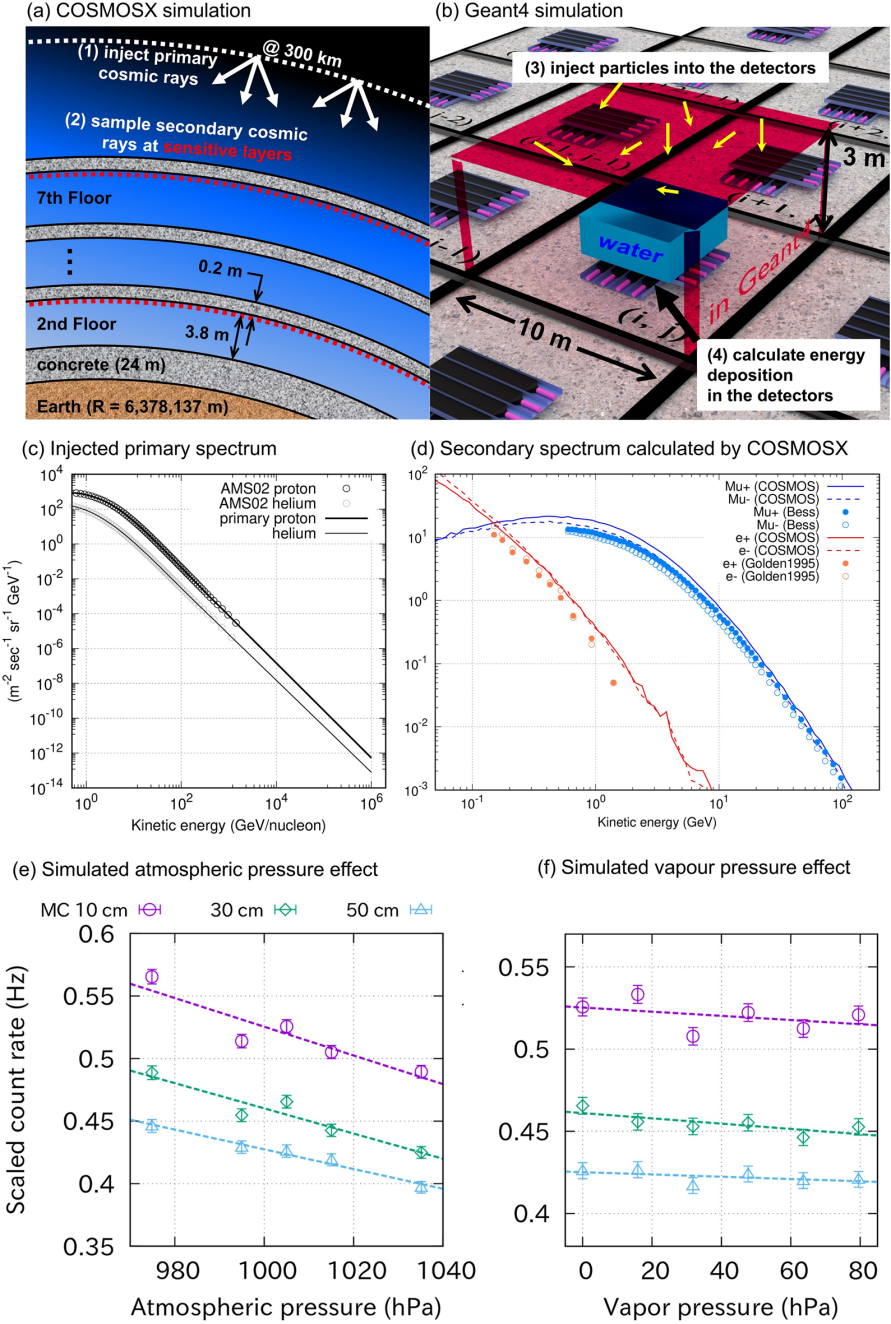
Setup of the two-step simulations and simulated effects of atmospheric pressure and water vapour pressure. (**a**) Schematic illustration of the simulation of cosmic-ray development in the atmosphere by COSMOSX simulator. (**b**) Schematic illustration of the simulation of the water pool and the detector response using Geant4 simulator. (**c**) Energy spectra of primary protons and helium nuclei taken from AMS-2 experiment. In COSMOSX simulation, protons and helium nuclei are injected following these spectra. (**d**) Energy spectra of secondary muons and electrons sampled at the sensitive layer at 50 m altitude in COSMOSX simulator (1005 hPa). The results are compared with literature values by Refs.^57,58^. (**e**) Simulated count rate depending on the atmospheric pressure (Colour representing different water levels in the tank). (**f**) Simulated count rate depending on the water vapour pressure.

The present study revealed significant atmospheric effects on the observed EM count rate. The primary effect is from atmospheric pressure ${\alpha }_{P}= -0.088\pm 0.008 (\%/{\text{hPa}}),$ followed by the water vapour pressure effect ${\alpha }_{V}= -0.019\pm 0.007 (\%/{\text{hPa}})$. The first effect is due to the atmosphere acting as an absorber for cosmic rays, which has been reported in many prior studies in terms of muon measurements^4–6^. The present study reports, for the first time, the minor effect of the atmospheric water content. This effect is probably due to the increase of < Z/A > (the weighted mean of the atomic number Z over mass number A) value of the atmosphere, and thus the stopping power for the charged particles becomes large^36,38^. In the regression analysis of this study, there may be additional minor uncertainty arising from the non-vapour water content in the clouds. According to a climatology study^45^, the major component of non-vapour water in the clouds of mid-latitude regions is in the form of liquid drops. The typical value of the vertically-integrated liquid water content in rainy clouds is approximately 2 kg m^−2^ (Ref.^46^). This indicates that the effect of the non-vapour water content is no more than that of 0.2 hPa variation in water vapour pressure and could be negligible (Fig. 7f).

In the present study, we tried a new approach for soil moisture measurements by employing a radiographic use of EM showers in cosmic rays. We have termed this new method as cosmic electromagnetic particle (CEMP) radiography. Here, we compared CEMP radiography with existing geophysical applications of cosmic rays: muography and neutron measurements. Among the numerous studies on muography, Jourde et al*.*^5^ performed an experiment similar to our water tank test. They installed a scintillation-type muography detector under a water tower in which the water level changed over time. They concluded that the muon count rate is modulated both by the atmospheric pressure and the water level with the corresponding coefficients of − 0.12%/hPa and − 0.09%/cm, respectively. The sensitivity of the EM count rate to the water level is 0.6–0.7%/cm in our calibration experiment, implying that CEMP radiography has 6–8 times higher sensitivity to the case where muons are employed as probes. However, it should be noted that count rates differ in muography and CEMP radiography by an order of magnitude when the detector sizes are the same.

CEMP radiography is compared to soil moisture measurement methods using cosmic-ray neutrons, which have recently gained popularity with several works^30–32^. This method prepares detectors for epithermal neutrons approximately 1–2 m above the soil and captures neutrons produced in the soil by cosmic rays. The intensity of the observed neutrons reflects the abundance of water in the soil because epithermal neutrons are greatly decelerated by hydrogen atoms in water. The most significant difference between EM and neutron methods is their coverage. While the neutron method is sensitive to the underlying soil moisture content of 130–240 m in radius^31^, CEMP radiography is sensitive to a localised change in soil moisture content just above the detector. In the case of neutron detectors in boreholes or buried beneath the snowpack^29^, the coverage might be close to that of CEMP radiography. Although the resolution of soil moisture content derived from the present work is poorer than neutron methods, CEMP radiography could provide complementary information to these existing methods.

The accuracy of soil moisture measurements by CEMP radiography could be further improved, for instance, by installing multiple detectors inside and above the tunnel. Such a configuration allows us to compensate for the atmospheric effects in real-time, and then the soil moisture content could be purely extracted. A method of calibration could also be advanced by performing an experiment where a large box of sand would be gradually filled with water. Another possible test is to repeat the study in or under the snowpack as was performed with neutron methods^29^. These technological developments would be necessary for quantitative monitoring. In the future, we envision that CEMP radiography could be used to monitor the occurrence of landslides, where an increase in fluid pressure inside the soil is expected before the collapse.

## Methods

### Detection system

The detection system (Fig. 2) consists of eight plastic scintillation planes (100 × 20 × 2 cm, produced by CI Industry^47^), eight photomultiplier tubes (Hamamatsu, H7724), and a custom 8 channel 80 MHz FADC board. The FADCs have a 12-bit resolution and a 0 to − 200 mV input voltage range. The analogue outputs from the PMTs are sent to the board. The board generates a trigger signal when four or more analogue signals out of eight channels exceed a certain threshold voltage within 500 nsec. When a trigger is generated, the FADC board sends the waveform integrals of all the eight channels to a local small Linux computer. The data were stored as an ASCII text file on the computer and transferred to the laboratory via a WiFi mobile network. The high-voltage for the PMTs (1.8 kV nominal, constant during the measurement) was generated by HAR-2N150 (Matsusada Precision Inc.) and divided into eight PMTs. Signal attenuators were inserted after the PMT outputs to make it possible to manually manipulate the effective gain of each PMT. This reduces the inherent variation in PMT gains. The applied high voltage, attenuation rate, and threshold voltage for trigger generation were adjusted manually by trial and error.

### Monte Carlo simulation

The objective of the MC simulation was to calculate the count rates of the events for different water levels in the water pool calibration test and for different atmospheric models. The simulation is performed in two steps.

(Step 1) The first step is to simulate the development of cosmic-ray showers in the atmosphere and building using the COSMOSX simulator (version 0.07)^48,49^. In the COSMOSX simulator, Earth is implemented as a sphere with a radius of 6,378,137 m. We constructed seven layers of 20-cm-thick concrete on the surface of the Earth to replicate the building. As illustrated in Fig. 7a, the horizontal dimension of the building is neglected, so the concrete floors (consisting of SiO_2_ with a density of 2.0 g/cm^3^) spread around the entire Earth. Primary cosmic rays (protons and helium nuclei) were injected into a sphere at 300 km of height. The injected nucleus, kinetic energy, position, and direction were randomly chosen from the primary cosmic-ray spectrum interpolated from the AMS-02 experiment^50^ below 1 TeV and extrapolated above 1 TeV (Fig. 7c). The spatial and temporal development of the injected primary was traced and stored when the particles crossed the sensitive layer attached to the ceiling of each floor. This sensitive layer was divided into rectangular meshes (periodical boundary with 10 × 10 m by sides, Fig. 7b), and further simulation was performed in the Geant4 simulator^51^. Atmospheric density and temperature were taken from the U.S. Standard Atmosphere^52^. This model was modified to evaluate the effects of (i) atmospheric pressure and (ii) water vapour pressure. Modification of the model is as follows: (i) the atmospheric density is uniformly multiplied by 0.97, 0.99, 1 = normal, 1.01, 1.03; (ii) the weight mass percentage of water is set to 0% = normal, 1%, 2%, 3%, 4%, 5%; hence, the mean molecular mass and the scale height are modified and the height of the troposphere was adjusted accordingly so that the ground (h = 50 m) pressure was kept constant (1005 hPa). In the simulation (ii), the effect of liquid water in the clouds was evaluated as that of the equivalent amount of water vapour under the assumption that the typical size of water drops in the clouds (10 mm) is negligibly smaller than the radiation length of liquid water (36 cm).

(Step 2) The next step was to simulate the effect of the water pool and detector response using the Geant4 simulator. In each cell, the detector (1 × 1 m by sides, Fig. 7b) and water tank were placed in the middle. Particles were injected from the ceiling using the information stored by the sensitive layers in the COSMOSX. After injection, the energy deposition in each of the eight plastic scintillation planes and the arrival time was stored. A hit is judged when the total energy deposition is greater than 2 MeV for four or more planes out of eight, and the entire event occurs within 500 nsec.

The energy spectra of the EM particles and muons simulated by COSMOSX are shown in Fig. 1 (Step 1). This calculation takes approximately 3.2 × 10^4^ thread hours using the Oakforest-PACS supercomputing system at the University of Tokyo^53^. The agreements between the COSMOSX-derived energy spectrum and the literature values were confirmed (Fig. 7d). General performance of COSMOSX simulation is confirmed by comparison with CORSIKA simulator^54^ and by a more intensive crosscheck with existing energy spectrum measurements for other particles^55^. According to Fig. 7d, the calculated spectra of the secondary particles are slightly higher than the literature values. This is probably because geomagnetic cut-off and solar modulation were not considered in the simulation^55^. Figure 3a shows the results of the Geant4 simulation (Step 2) for the 2nd and 7th floors for different water levels considered. The decreasing trend of the EM event is generally reproduced, although the absolute rate differs by a factor of 1.8 (observation > simulation). The difference may be because the hadronic interaction models employed in COSMOSX are not sufficiently accurate; the transverse momentum may be overestimated or the multiplicity of particle production may be underestimated. Figure 7e,f show the results of changing the atmospheric density (atmospheric pressure) and the water content in the atmosphere (water vapour pressure).

## Supplementary Information

Supplementary Information.

## Data Availability

The count-rate data collected in this study is available at the supplementary file. Other data/information are available from the corresponding author upon requests. The meteorological data is available at JMA website https://www.data.jma.go.jp/gmd/risk/obsdl/index.php.

## Code availability

Geant4 https://geant4.web.cern.ch/. COSMOSX http://cosmos.icrr.u-tokyo.ac.jp/COSMOSweb/.

## References

[1] Dorman LI (2004). Cosmic Rays in the Earth’s Atmosphere and Underground.

[2] Grieder PK (2001). Cosmic rays at Earth.

[3] Cecchini S, Spurio M (2012). Atmospheric muons: Experimental aspects. Geosci. Instrum. Method Data Syst..

[4] Pierre Auger Collaboration (2011). The Pierre Auger Observatory scaler mode for the study of solar activity modulation of galactic cosmic rays. J. Inst..

[5] Jourde K (2016). Monitoring temporal opacity fluctuations of large structures with muon radiography: A calibration experiment using a water tower. Sci. Rep..

[6] De Mendonça RRS (2011). Long-term and transient time variation of cosmic ray fluxes detected in Argentina by CARPET cosmic ray detector. J. Atmos. Sol. Terr. Phys..

[7] Borexino collaboration, Cosmic-muon flux and annual modulation in Borexino at 3800 m water-equivalent depth., *J. Cosmol. Astropart. P.*, **2012**, 015, 10.1088/1475-7516/2012/05/015 (2012).

[8] GERDA collaboration, Flux modulations seen by the muon veto of the GERDA experiment. *Astropart. Phys.*, **84**, 29–35, 10.1016/j.astropartphys.2016.08.002 (2016).

[9] Tramontini M, Rosas-Carbajal M, Nussbaum C, Gibert D, Marteau J (2019). Middle-atmosphere dynamics observed with a portable muon detector. Earth Space Sci..

[10] Tanaka HKM (2021). First results of undersea muography with the Tokyo-Bay Seafloor hyper-kilometric submarine deep detector. Sci. Rep..

[11] Tanaka HKM, Nakano T, Takahashi S, Yoshida J, Niwa K (2007). Development of an emulsion imaging system for cosmic-ray muon radiography to explore the internal structure of a volcano, Mt. Asama. Nucl. Instrum. Methods Phys. Res. A.

[12] Oláh L, Tanaka HKM, Ohminato T, Varga D (2018). High-definition and low-noise muography of the Sakurajima volcano with gaseous tracking detectors. Sci. Rep..

[13] Lesparre N (2012). Density muon radiography of La Soufrière of Guadeloupe volcano: Comparison with geological, electrical resistivity and gravity data. Geophys. J. Int..

[14] Le Gonidec Y (2019). Abrupt changes of hydrothermal activity in a lava dome detected by combined seismic and muon monitoring. Sci. Rep..

[15] Ambrosi G (2011). The MU-RAY project: Volcano radiography with cosmic-ray muons. Nucl. Instrum. Methods Phys. Res. A.

[16] Nishiyama R, Miyamoto S, Okubo S, Oshima H, Maekawa T (2017). 3D density modelling with gravity and muon-radiographic observations in Showa-Shinzan Lava Dome, Usu Japan. Pure Appl. Geophys..

[17] Presti Lo (2020). Muographic monitoring of the volcano-tectonic evolution of Mount Etna. Sci. Rep..

[18] Ambrosino F (2015). Joint measurement of the atmospheric muon flux through the Puy de Dôme volcano with plastic scintillators and Resistive Plate Chambers detectors. J. Geophys. Res. Solid Earth.

[19] Tioukov V (2019). First muography of Stromboli volcano. Sci. Rep..

[20] Nishiyama R (2017). First measurement of ice-bedrock interface of alpine glaciers by cosmic muon radiography. Geophys. Res. Lett..

[21] Tanaka HKM (2011). Cosmic muon imaging of hidden seismic fault zones: Rainwater permeation into the mechanical fractured zones in Itoigawa-Shizuoka Tectonic Line, Japan. Earth Planet Sc. Lett..

[22] Yamazaki K, Taketa A, Ikeda D, Omura K (2022). Development of detector and method for density structure measurement of fault zones using cosmic ray muons. Nucl. Instrum. Methods Phys. Res. A..

[23] Schouten DW, Ledru P (2018). Muon tomography applied to a dense uranium deposit at the McArthur River mine. J. Geophys. Res. Solid Earth.

[24] Morishima K (2017). Discovery of a big void in Khufu’s Pyramid by observation of cosmic-ray muons. Nature.

[25] The IceCube Collaboration (2017). Measurement of the multi-TeV neutrino interaction cross-section with IceCube using earth absorption. Nature.

[26] Rott C, Taketa A, Bose D (2015). Spectrometry of the earth using neutrino oscillations. Sci. Rep..

[27] Kodama M (1980). Continuous monitoring of snow water equivalent using cosmic ray neutrons. Cold Reg. Sci. Technol..

[28] Howat IM, de la Peña S, Desilets D, Womack G (2018). Autonomous ice sheet surface mass balance measurements from cosmic rays. Cryosphere.

[29] Gugerli R, Salzmann N, Huss M, Desilets D (2019). Continuous and autonomous snow water equivalent measurements by a cosmic ray sensor on an alpine glacier. Cryosphere.

[30] Evans JG (2016). Soil water content in southern England derived from a cosmic-ray soil moisture observing system—COSMOS-UK. Hydrol. Process.

[31] Köhli M (2015). Footprint characteristics revised for field-scale soil moisture monitoring with cosmic-ray neutrons. Water Resour. Res..

[32] Stevanato L (2019). A novel cosmic-ray neutron sensor for soil moisture estimation over large areas. Agriculture.

[33] Niedermann S (2002). Cosmic-ray-produced noble gases in terrestrial rocks: Dating tools for surface processes. Rev. Mineral. Geochem..

[34] Mair D (2019). Fast long-term denudation rate of steep alpine headwalls inferred from cosmogenic ^36^Cl depth profiles. Sci. Rep..

[35] Tsuchiya H (2007). Detection of high-energy gamma rays from winter thunderclouds. Phys. Rev. Lett..

[36] Zyla PA (2020). Review of particle physics. Prog. Theor. Exp. Phys..

[37] Berger MJ, Coursey JS, Zucker MA, Chang J (2017). Stopping-power and range tables for electrons, protons and helium ions. NISTIR.

[38] Groom DE, Mokhov NV, Striganov SI (2002). Muon stopping power and range tables 10 MeV–100 TeV. At. Data Nucl. Data Tables.

[39] Murata H, Okabayashi T (1983). The erosion and failure of the volcanic ash slopes of Sakurajima. Technol. Rep. Yamaguchi Univ..

[40] Japan Meteorological Agency. https://www.jma.go.jp/jma/indexe.html. Accessed 14 Oct 2002.

[41] Kazama, T., Hydrological modeling of groundwater disturbances to observed gravity data toward high-accuracy monitoring of magma transfer in volcanoes, Doctoral thesis, The University of Tokyo, 10.15083/0002003368 (2010). In Japanese.

[42] Teramoto Y (2018). Effect of volcanic activity on succession of woody vegetation and water infiltration rates on peripheral slopes of Sakurajima Volcano Japan. J. Rainwater Catchment Syst..

[43] Kazama T, Okubo S (2009). Hydrological modeling of groundwater disturbances to observed gravity: Theory and application to Asama Volcano, Central Japan. J. Geophys. Res..

[44] Imanishi Y, Kokubo K, Tatehata H (2006). Effect of underground water on gravity observation at Matsushiro, Japan. J. Geodyn..

[45] Kida S, Shige S, Manabe T, L’Ecuyer T, Liu G (2010). Cloud liquid water path for the Rain/No-rain classification method over ocean in the GSMaP algorithm. Trans. JSASS Aerosp. Tech. Japan.

[46] Dong C, Weng F, Yang J (2022). Assessments of cloud liquid water and total precipitable water derived from FY-3E MWTS-III and NOAA-20 ATMS. Remote Sens..

[47] Yamashina Y, Yamashita T, Taira H, Tanaka HKM (2010). Development of a cost-effective plastic scintillator for cosmic-ray muon radiography of a volcano. Earth Planets Space.

[48] Sako, T. *et al.*, COSMOS X as a general purpose air shower simulation tool, *ICRC2021*, 431; 10.22323/1.395.0431 (2021)

[49] CosmosX Air Shower MC Tool. http://cosmos.icrr.u-tokyo.ac.jp/COSMOSweb/. Accessed 14 Oct 2022.

[50] Aguilar M, AMS Collaboration (2015). Precision measurement of the proton flux in primary cosmic rays from rigidity 1 GV to 1.8 TV with the alpha magnetic spectrometer on the international space station. Phys. Rev. Lett..

[51] Allison J (2016). Recent developments in Geant4. Nucl. Instrum. Methods Phys. Res. A.

[52] U.S. Government Printing Office, *U.S. Standard Atmosphere*, Washington, D.C. (1976).

[53] Supercomputing Division, Information Technology Center, The University of Tokyo. https://www.cc.u-tokyo.ac.jp/en/. Accessed 14 Oct 2022.

[54] Roh S (2013). A comparison study of CORSIKA and COSMOS simulations for extensive air showers. Astropart. Phys..

[55] Nishiyama R, Taketa A, Miyamoto S, Kasahara K (2016). Monte Carlo simulation for background study of geophysical inspection with cosmic-ray muons. Geophys. J. Int..

[56] Wessel, P., Smith, W. H. F., Scharroo, R., Luis, J. & Wobbe, F., Generic Mapping Tools: Improved Version Released, *EOS Trans. AGU*, **94**(45), 409–410; 10.1002/2013EO450001 (2013). Software available at https://www.generic-mapping-tools.org/.

[57] Haino S (2004). Measurements of primary and atmospheric cosmic-ray spectra with the BESS-TeV spectrometer. Phys. Lett. B.

[58] Golden RL (1995). Measurement of the energy spectra of cosmic ray electron component and protons at ground level. J. Geophys. Res-Space..

